# Isolated metastasis of colon cancer to the scapula: is surgical resection warranted?

**DOI:** 10.1186/1477-7819-9-137

**Published:** 2011-10-26

**Authors:** Jill K Onesti, Christopher R Mascarenhas, Mathew H Chung, Alan T Davis

**Affiliations:** 1 GRMEP/MSU General Surgery Residency Program, 1000 Monroe Ave.NW, Grand Rapids, MI, 49503, USA; 2 Michigan State University Department of Surgery, Sparrow Professional Building, 1200 E. Michigan Ave., Lansing, MI, 48912, USA

**Keywords:** colon cancer, metastasis, scapula

## Abstract

**Background:**

Distant metastases from colon cancer spread most frequently to the liver and the lung. Risk factors include positive lymph nodes and high grade tumors. Isolated metastases to the appendicular skeleton are very rare, particularly in the absence of identifiable risk factors.

**Case report:**

The patient was a 55 year old male with no previous personal or family history of colon cancer. Routine screening revealed a sigmoid adenocarcinoma. He underwent resection with primary anastomosis and was found to have Stage IIA colon cancer. He declined chemotherapy as part of a clinical trial, and eight months later was found to have an isolated metastasis in his right scapula. This was treated medically, but grew to 12 × 15 cm. The patient underwent a curative forequarter amputation and is now more than four years from his original colon surgery.

**Discussion:**

Stage IIA colon cancers are associated with a high five year survival rate, and chemotherapy is not automatically given. If metastases occur, they are likely to arise from local recurrence or follow lymphatic dissemination to the liver or lungs. Isolated skeletal metastases are quite rare and are usually confined to the axial skeleton. To our knowledge, this is the first reported case of an isolated scapular metastasis in a patient with node negative disease. The decision to treat the recurrence with radiation and chemotherapy did not reduce the tumor, and a forequarter amputation was eventually required.

**Conclusion:**

This case highlights the importance of adequately analyzing the stage of colon cancer and offering appropriate treatment. Equally important is the early involvement of a surgeon in discussing the timing of the treatment for recurrence. Perhaps if the patient had received chemotherapy or earlier resection, he could have been spared the forequarter amputation. The physician must also be aware of the remote possibility of an unusual presentation of metastasis in order to pursue timely work up.

## Background

Distant metastases from colon cancer spread most frequently to the liver and the lung. Risk factors include high grade tumors and lymph node involvement. Isolated metastases to the appendicular skeleton are very rare, particularly in the absence of identifiable risk factors.

## Case report

The patient was a 55 year old male with iron deficiency anemia and no family history of colon cancer. His work up included an esophagogastroduodenoscopy and colonoscopy, which revealed a sigmoid adenocarcinoma and a questionable mass in the cecum. A computed tomography (CT) scan of his chest, abdomen and pelvis did not indicate additional disease. His carcinoembryonic antigen (CEA) level was 1.6 ng/mL. In early 2007, the patient underwent a sigmoid resection and right hemicolectomy with primary anastomosis. The final pathology confirmed a diagnosis of T3N0M0 adenocarcinoma with seven negative lymph nodes in the sigmoid colon and a benign polyp in the cecum.

After establishing the diagnosis of stage IIA sigmoid cancer, the patient was offered chemotherapy as part of a clinical trial, but declined. At three months he noticed right shoulder pain, and at five months he noticed a small, golf-ball sized mass. Eight months after his surgery, he presented to his primary care physician who immediately referred him to an orthopedic surgeon. His initial magnetic resonance imaging (MRI) demonstrated a 10 × 10 cm mass originating from his right scapula. A core needle biopsy was performed, revealing adenocarcinoma consistent with a colorectal primary. His CEA level at this point was 1480 ng/mL. A hybrid CT/PET (positron emission tomography) scan as well as additional lab work did not identify any additional focus of metastasis.

The patient and his doctors elected to treat the metastasis with chemotherapy and radiation. He was started on radiotherapy with radiosensitization using Capecitabine followed by chemotherapy with FOLFOX (infusional 5-fluorouracil, leucovorin, and oxaliplatin) and Bevacizumab. After 12 cycles, the patient developed significant side effects, and the regimen was changed to FOLFIRI (infusional 5-fluorouracil, leucovorin, and irinotecan).

**Figure 1:**
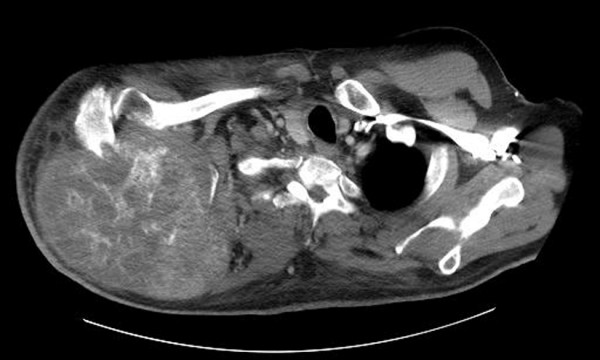
**Preoperative CT scan demonstrating large scapular mass**.

Serial CT and MRI scans during the chemotherapy demonstrated increasing size of the mass. Twenty months after his initial surgery, the patient noticed a decline in his arm function while his pain continued to increase. At this point, his MRI showed a 12 × 15 cm mass, his CEA level was 484 ng/mL and his alkaline phosphatase was 183 IU/L. Another hybrid CT/PET scan in late 2008 (Figure 1) again demonstrated no additional focus of metastasis, but did show close proximity of the tumor to the chest wall.

**Figure 2:**
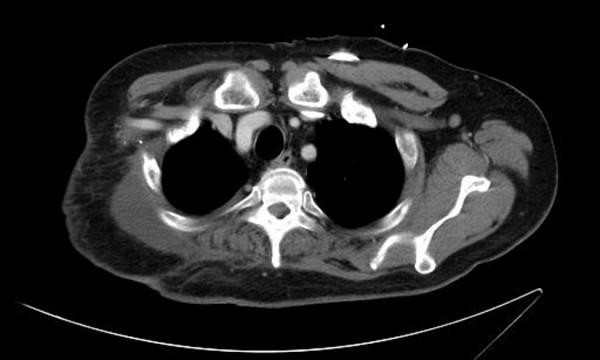
**Post-operative CT scan showing the intact chest wall and removal of tumor**.

The patient's oncologist referred him to our center, and forequarter amputation with possible chest wall reconstruction was recommended. A month after the CT/PET scan, the surgical oncologist removed the patient's right arm and scapula, but was able to avoid resecting the chest wall (Figure 2). A plastic surgeon provided reconstruction using a musculocutaneous flap. Final pathology demonstrated negative margins. The patient healed well without incident and is working with physical therapy. Six months following the surgery, his CEA level was 1.6 ng/mL and has remained low on subsequent follow-up visits. The most recent information is from mid-2011, where the CEA level was determined to be 1.5 ng/mL, and a CT was negative for any evidence of metastasis.

## Discussion

Colorectal cancer is the third most common cause of cancer in men and women in the United States [1]. Screening for colon cancer is an important factor in decreasing the morbidity and mortality of the disease, given the much higher survival rate for earlier stage tumors versus more advanced disease. Stage IIA colon cancer (T3N0M0 lesions) has a high five year survival rate of 85% as reported by the SEERS database [2]. Given the excellent survival rate based on surgery alone, medical treatment of Stage II colon cancers remains somewhat controversial. The National Comprehensive Cancer Network (NCCN) has examined several studies and currently recommends either chemotherapy, enrollment in a clinical trial, or close observation after adequate surgical excision for Stage IIA cancers [3]. The NCCN also identified several risk factors that may help determine which patients would benefit most from the addition of chemotherapy. These include high grade tumor characteristics on histology, perforation or obstruction at the time of presentation, lymphovascular invasion, or fewer than 12 lymph nodes removed during resection. A single risk factor was identified in this patient in that only seven nodes were resected, but he declined initial chemotherapy.

Skeletal metastases with colon cancer occur in 5-10% of cases and usually after widespread metastatic disease [4-8]. The usual pattern of metastasis is through the liver and the lungs, thus making isolated skeletal metastases even more uncommon, at 1-2% [4,6]. The most likely route for skeletal seeding is through Batson's plexus, a valveless system of veins draining to the vertebral column, making it the most common site for skeletal metastasis. Other frequent sites include the skull, pelvis, femur and humerus [4,9-11]. Unusual sites have been reported in the literature, but to our knowledge, this is the first report of an isolated scapular metastasis in a patient with negative lymph nodes.

Successful treatment of scapular tumors correlates with complete resection [12], and served as the impetus for surgical selection in this case. While a forequarter amputation is associated with significant cosmetic, functional, and psychological impacts, it is a valuable procedure to achieve negative margins in advanced tumors and may aid in significant palliation [13]. Historically there are two well defined techniques to consider when performing a forequarter amputation. These include the anterior (Berger) approach and the posterior (Littlewood) approach. Our technique consisted of a combined anterior and posterior approach as described by Ferrario et al. [14], allowing complete dissection of bone and soft tissue prior to transection of the subclavian vessels. This approach provides circumferential access to the vessels and brachial plexus prior to ligation and significantly reduces the risk of uncontrolled bleeding as well as allowing for a greater proximal margin.

Perhaps if the tumor had been discovered earlier, it may have been amenable to a limb-sparing scapulectomy. A complete scapulectomy or Tikhoff-Linberg procedure is associated with a significant complication rate, but does provide an acceptable treatment for shoulder cancers if negative margins are achieved [15]. Recovery from scapulectomy involves therapy and psychological adjustment, but has an overall favorable report [16]. A limb-sparing surgery was not adequate in this case according to published guidelines, given the extensive size of the tumor and possible chest wall invasion which would have made achieving an R0 resection unlikely [17,18].

Difficulty often arises in determining optimal timing and course of treatment for unusual metastases. A multi-disciplinary team reviewed this patient's case and agreed to first pursue chemotherapy and radiation rather than resection. It is possible that the patient's upper extremity could have been spared had the resection occurred earlier. While chemotherapy does remain a mainstay treatment of metastatic colon cancer, if excision of a solitary lesion offers the potential of an R0 resection, it is generally advised. Earlier treatment may also have avoided preoperative radiation. While advances have been made in approaching the reconstruction of irradiated tissues [19,20], chest wall resection and reconstruction (if necessary) would have been much simpler prior to receiving radiation.

## Conclusion

This case highlights the importance of adequately analyzing the stage of colon cancer (with adequate lymph node resection) and offering appropriate treatment. Equally important is the early involvement of a surgeon in discussing the timing of the treatment for recurrence and metastasis. Perhaps if the patient had initially received chemotherapy or earlier resection of the recurrence, he could have been spared the forequarter amputation. The physician must also be aware of the remote possibility of an unusual presentation of metastasis in order to pursue timely work up. Fortunately, this patient is now three years from his amputation and over four years from his initial sigmoid resection. He continues to do well with physical therapy and is maintaining close follow up with no evidence of disease.

## Consent

Written informed consent was obtained from the patient for publication of this case report and accompanying images. A copy of the written consent is available for review by the Editor-in-Chief of this journal.

## Competing interests

The authors declare that they have no competing interests.

## Authors' contributions

JKO, CRM and MHC participated in the admission and the care of this patient. All the authors participated in the conception, the design, data collection and interpretation, manuscript preparation and literature search. All authors have read and approved the final manuscript.
